# Friendships and Family Support Reduce Subsequent Depressive Symptoms in At-Risk Adolescents

**DOI:** 10.1371/journal.pone.0153715

**Published:** 2016-05-04

**Authors:** Anne-Laura van Harmelen, Jenny L. Gibson, Michelle C. St Clair, Matt Owens, Jeannette Brodbeck, Valerie Dunn, Gemma Lewis, Tim Croudace, Peter B. Jones, Rogier A. Kievit, Ian M. Goodyer

**Affiliations:** 1 Department of Psychiatry, University of Cambridge, Cambridge, United Kingdom; 2 Faculty of Education, University of Cambridge, Cambridge, United Kingdom; 3 Department of Psychology, University of Bath, Bath, United Kingdom; 4 Department of Psychology, University of Exeter, Exeter, United Kingdom; 5 Department of Psychology, University of Berne, Berne, Switzerland; 6 School of Nursing and Health Sciences, University of Dundee, Dundee, Scotland, United Kingdom; 7 Medical Research Council, Cognition and Brain Sciences Unit, Cambridge, United Kingdom; West Virginia University School of Medicine, UNITED STATES

## Abstract

**Background:**

Early life stress (ELS) consists of child family adversities (CFA: negative experiences that happened within the family environment) and/or peer bullying. ELS plays an important role in the development of adolescent depressive symptoms and clinical disorders. Identifying factors that may reduce depressive symptoms in adolescents with ELS may have important public mental health implications.

**Methods:**

We used structural equation modelling and examined the impact of adolescent friendships and/or family support at age 14 on depressive symptoms at age 17 in adolescents exposed to ELS before age 11. To this end, we used structural equation modelling in a community sample of 771 adolescents (322 boys and 477 girls) from a 3 year longitudinal study. Significant paths in the model were followed-up to test whether social support mediated or moderated the association between ELS and depressive symptoms at age 17.

**Results:**

We found that adolescent social support in adolescence is negatively associated with subsequent depressive symptoms in boys and girls exposed to ELS. Specifically, we found evidence for two mediational pathways: In the first pathway family support mediated the link between CFA and depressive symptoms at age 17. Specifically, CFA was negatively associated with adolescent family support at age 14, which in turn was negatively associated with depressive symptoms at age 17. In the second pathway we found that adolescent friendships mediated the path between peer bullying and depressive symptoms. Specifically, relational bullying was negatively associated with adolescent friendships at age 14, which in turn were negatively associated with depressive symptoms at age 17. In contrast, we did not find a moderating effect of friendships and family support on the association between CFA and depressive symptoms.

**Conclusions:**

Friendships and/or family support in adolescence mediate the relationship between ELS and late adolescent depressive symptoms in boys and girls. Therefore, enhancing affiliate relationships and positive family environments may benefit the mental health of vulnerable youth that have experienced CFA and/or primary school bullying.

## Introduction

Adolescence is a key developmental time where the incidence and prevalence of mental illnesses such as major depression (MD) increases considerably[1]. It is well established that exposure to negative experiences within the family environment (childhood family adversities; CFA) increases risk for depressive symptomatology[2–4]. CFA may include negative parenting styles, emotional, physical, sexual abuse, lack of affection or engagement, family discord, financial problems, family loss, criminality, unemployment, and parental psychopathology. Parent child interactions set the stage for later peer interactions (see Pallini, Baiocco, Schneider, Madigan, & Atkinson, 2014 for a meta-analysis). Indeed, CFA increases risk for (chronic) bullying from peers[6–13]. One way through which CFA can increase risk for peer bullying is through increased arousal and anxiety [14,15]which may be adaptive in negative family environments but may be maladaptive in the outside world[16].

Bullying by peers is a similar toxic social experience that has been associated with subsequent mental illness (e.g.[17–20]), including increased risk for, and chronicity of, depressive disorders (e.g.[21–26], with effects possibly being stronger in boys[27], although others did not find gender effects[21]). Peer bullying may represent a key link between CFA and later depressive symptoms[28], at least in children from low socio-economic backgrounds[29]. Importantly, the combined experience of CFA *and* peer bullying has been associated with increased severity of depression symptoms[30]. Adolescent depression predicts a cascade of behavioural and mental health problems, including recurrent depressive disorders and depression is a major risk for suicide in adolescents [1]. Therefore, in order to reduce adolescent depression, it is crucial to identify environmental factors that may increase resilience in adolescents who have experienced CFA and/or peer bullying

As childhood progresses into adolescence, social environments widen and friendships become increasingly important for social, and psychological development[31,32]. For this reason, friendship support may be an especially important factor that may increase resilience in vulnerable adolescents[33]. Several studies have examined the impact of adolescent friendships on depressive symptoms in adolescents who have been exposed to CFA and/or peer bullying, with mixed findings. For instance, in a cross-sectional study, adolescent friendships have been associated with lower likelihood of depressive symptoms in boys[34]. Furthermore, adolescent friendships were associated with higher rates of later life resilience (defined as the absence of adult psychopathology over a 30 year time-period) in a small sample of abused individuals [35].

Supportive *family* environments may be another important social factor that may increase adolescent resilience after CFA and/or peer bullying[33]. Studies that examined the impact of positive parenting on depression in adolescents that reported CFA/ peer bullying indicate differential findings. Abused individuals that reported at least one parent as caring in adolescence had higher rates of adult resilience (defined as the absence of adult psychopathology over a 30 year time-period[35]). In line with these findings, adolescent bullying was associated with increases in later adolescent depression, but only in those adolescents without supportive parents[36]. Similarly, more positive parental quality was associated with reduced association between peer stress (including bullying) and depressive symptoms[37]. Furthermore, maternal warmth between ages 5–10 has been found to reduce the relationship between peer bullying in primary school and emotional problems at age 10[38]. However, others only found that, in a cross sectional design, supportive parents may reduce mental health difficulties in victimized adolescent girls, but not in boys[39]. Finally, supportive parenting accounted for greater variance than CFA in depressive symptoms in adolescents[40]. Null effects were also reported in the cross-sectional relationship between supportive parenting and peer bullying in boys[34], and parental verbal affection did not mediate the relationship between CFA before the age of 6, and psychiatric symptoms [41].

In sum, there are indications that adolescent friendships and supportive family environments may increase resilience in adolescents who have experienced peer bullying and/or CFA, although there may be gender differences. However, these studies examined either friendship support [37,42,43], or family support[38,40,44] in isolation, and unmeasured co-occurrence between friendships and family support (e.g.[5,45]) may hamper the interpretability of these findings. Other studies investigated CFA[40] or peer bullying[34] in isolation, despite the fact that these two negative experiences co-occur frequently (e.g.[7]). A recent study in adolescents aged 10–17 showed that current family support and friendship support were not related to current distress after recent (past 2 years) poly-victimization (including bullying and CFA)[46]. However, this study did not examine early life CFA, nor peer bullying, nor did they disentangle the specific relations of CFA and peer bullying with friendships and family support. Thus, to our knowledge, no study has simultaneously examined the interplay of early life CFA and/or peer bullying, and family support and/or friendships in adolescence on later adolescent depressive symptoms.

Our study uses Structural Equation Modelling (SEM) in a longitudinal population based community sample (N = 771; 322 boys and 477 girls) to examine the relations between CFA and peer bullying before age 11, family and friendships at age 14, and depressive symptoms at age 17. In addition, we also examine whether we find any evidence for gender-specific effects. We expected that the social environment mediates the association between CFA/peer bullying and depressive symptoms as peer bullying adversely affects future peer interactions[47,48], and friendship difficulties increases depressive symptoms[32]. Furthermore, a negative family environment is relatively persistent throughout childhood [49], and it is likely that CFA is associated with reduced family support in adolescence. Therefore, we choose SEM testing these hypothesized mediation effects of friendship and/or family support after CFA and/or peer bullying. Furthermore, significant paths in our model were followed up with specific mediation analyses. In addition, as a moderating (‘Buffering’) hypothesis of social support has also been suggested[33], we also tested whether we found support for such moderations (i.e. whether social support reduces the association between CFA/peer bullying and depressive symptoms at age 17).

Finally, there are indications that depressive symptoms may form antecedents to negative peer relations [24,50], thus it is vital that sequential events are examined when investigating the impact of the social environment on late adolescent depression. To examine the temporal dynamics of our findings, we also examined longitudinal effects of adolescent support on depressive symptoms in later life. We chose to use a cross-lagged SEM as this allowed us to simultaneously assess the effects of family support, depression symptoms, and friendships over time (from age 14 to 17). This also allows us to test the reverse hypothesis that higher depression symptoms at age 14 are associated with reduced friendships and family support at age 17, which could explain associations between family support and friendships at age 14 and depression symptoms at age 17.

## Materials and Methods

### Participants

Participants were drawn from the ROOTS study; a 3-year longitudinal study of adolescent development in 1238 participants from 18 Cambridgeshire secondary schools between November 2005 and January 2010, see[51,52] for more information on ROOTS. ROOTS was approved by the local Cambridge Research Ethics Committee [RNAG/360]. Written informed consent was obtained from both children and their caregivers. 771 adolescents (62.3%) had complete data on all measures for this investigation (Table 1). This subsample was not significantly different (*p* values>.10) from the full ROOTS cohort on: age, sex, socio-economic status, CFA, peer bullying, family support, friendships, and depressive symptoms at age 17.

**Table 1:** Sample characteristics.

Variables used	Total N (%) with complete data:	Descriptives for sample used N = 771 (62.3%)				
**Sex**	N = 1238 (100%)	322 (41.8%) Boys		449 (58.2%) Girls		
**Family adversity classes**	N = 1139 (92.0%)	558 (72.4%) Optimal	52 (6.7%) Aberrant	128 (16.6%) Discordant	33 (4.3%) Hazardous	
**Bullying**	**N = 900 (72.7%)**	**Never**	**Once**	**Sometimes**	**Weekly**	**Daily**
**I was hit, punched or kicked**	N = 920 (74.3%)	616 (79.9%)	72 (9.3%)	68 (8.8%)	11 (1.4%)	4 (0.5%)
**I was scratched**	N = 915 (73.9%)	685 (88.8%)	37 (4.8%)	42 (5.4%)	7 (0.9%)	0 (0%)
**I was threatened**	N = 915 (73.9%)	597 (77.4%)	54 (7.0%)	88 (11.4%)	26 (3.4%)	6 (0.8%)
**I was sent nasty notes/texts/emails**	N = 910 (73.5%)	681 (88.3%)	30 (3.9%)	52 (6.7%)	5 (0.6%)	3 (0.4%)
**I was ignored**	N = 925 (74.7%)	546 (70.8%)	48 (6.2%)	105 (13.6%)	38 (4.9%)	34 (4.4%)
**People said nasty things about me**	N = 927 (74.9%)	506 (65.6%)	62 (8.0%)	118 (15.3%)	56 (7.3%)	29 (3.8%)
**I felt unable to defend myself**	N = 921 (74.4%)	610 (79.1%)	33 (4.3%)	62 (8.0%)	37 (4.8%)	29 (3.8%)
**I was frightened**	N = 918 (74.2%)	590 (76.5%)	41 (5.3%)	85 (11.0%)	33 (4.3%)	22 (2.9%)
**Factor and sum scores**		**mean (SD)**	**Min**	**Max**		
**Relational bullying Factor score**	N = 900 (72.7%)	-1.46 (1.32)	-3.02	2.06		
**Physical bullying Factor score**	N = 900 (72.7%)	-0.98 (1.41)	-2.32	3.31		
**Friendships at age 14**	N = 1133 (91.5%)	23.54 (4.21)	2	30		
**Family support at age 14**	N = 1105 (89.3%)	-22.43 (5.76)	-48	-12		
**Depressive symptoms sum score at age 17**	N = 1007 (81.3%)	13.6 (10.21)	0	57		

Note. The total number of individuals with complete data per variable in ROOTS is depicted in column 2. There were no significant differences between these variables when comparing that total number (i.e. the ROOTS sample) with this subsample (N = 771). The factor scores depicted here were based on the ‘final model’ SEM with N = 771. All participants were 14 years of age upon entry to the study when childhood family adversities, peer, and family support were assessed. All participants were 17 years of age when we re-assessed depressive symptoms, and retrospectively assessed primary school peer bullying.

### Childhood Family Adversity (CFA)

CFA was assessed when the participants were aged 14 using the Cambridge Early Experiences Interview (CAMEEI)[49]. The CAMEEI is a semi-structured, respondent-sensitive, interviewer led procedure that collected retrospective accounts of the quality of family environment. These recalled experiences were obtained from the main caregiver independently of the self-reported assessments carried out with their adolescent offspring. The caregivers being interviewed were biological mothers (96%, N = 1143), biological fathers (3%, N = 35), adoptive mothers (N = 7), both parents (N = 3) and N = 2 each of extended family members, step-mothers and step-fathers. The CAMEEI focuses on three time domains of childhood: “early childhood” (preschool years–birth until approximately five years of age), “later childhood” (approximately six-11 years) and “early adolescence” (approximately 11 years– 14 years).

Adversities reported in the CAMEEI were: 1) Negative family relationships (family loss and separations (includes step parents and siblings and partners resident for more than 6 months) through divorce, death or adoption; ii) family discord; iii) lack of maternal affection/engagement with the proband; iv) maternal parenting style and v) paternal parenting style), family discord. 2); i) lifetime family medical illnesses sufficiently severe to impact on family life (moderate, chronic and life-threatening); ii) lifetime psychopathology in family members 3) Family Economics i) periods of unemployment; ii) financial difficulties. 4) Childhood Maltreatment: i) physical abuse; ii) sexual abuse; iii) emotional abuse. Including 'at risk' children defined as those ever having been on the Child Protection Register or for whom there was strong, but inconclusive, evidence of abuse. 5) Other Events; i) criminality among family members, ii) acute life events, and iii) chronic social difficulties (e.g. ongoing litigation or the demands of caring for extended family).

In previous work[49], we used latent class analysis to identify subgroups of adolescents who had experienced different types of early adversity, based on their CAMEEI data. Latent Class Analyses (LCA) assumes that a population can be divided into mutually exclusive and exhaustive latent groups (classes) based on individual response patterns from a set of measured items[53]. Identifying these latent classes is of value because different groups have different characteristics, different prognoses and therefore different aetiologies. We found support for four mutually exclusive CFA subgroups[16]. The largest class (the ‘Optimal class’) contained those with a low (<13%) probability of any adversity at any time-point (n = 784, 69% of the sample). The second (‘Abberant Parenting’; n = 76, 7% of the sample) had a high probability (70–100%) of inconsistent and atypical parenting by both parents (e.g. lax, very strict, cruel to be kind, hitting, all of which showed low prevalence) and a lower probability (8–17%) of any adversity at any time-point. The third class (the ‘Discordant class’; n = 213, 19% of the sample) had a high probability (47%) of family discord (e.g. marital disagreements) and a 11–39% probability of any adversity at any time-points. They also showed elevated rates of family loss, financial difficulties, and maternal psychiatric illness. The fourth class (the ‘Hazardous class’; n = 66, 6% of the sample) had 50–90% probability of any adversity at any time-point) with a high probability (60%) of physical and/or emotional abuse. These classes were replicated at each time-point (birth to age 5; ages 6 to 11; and ages 12 to 14) [44]. In this study, we focused on adversities in the preschool years (birth-5), as these adversity classes have been shown to be relatively stable and thus represent persistent exposure to a suboptimal family environment[49]. We used the four CFA latent classes that were based on our previous report[49]. Given the fact that some of these classes are relatively small, and to ensure the robustness of our findings, we also reran the model with a dichotomous CFA division comparing an ‘Optimal’ vs. ‘Adverse’ (i.e. the Discordant, Hazardous, and Aberrant classes combined) childhood. We found that the findings were virtually identical. Note that all results in the current study also remained the same when the analyses were performed with the classes at age six-11.

### Primary school peer bullying

We assessed primary school (age five-11) peer bullying using the self-report Peer Victimization Questionnaire (PVQ, see S1 Appendix). The PVQ was completed retrospectively when participants were aged 17. Average split-half reliability in our sample was good α = .91 (95% CI = .89-.92). As this is a new scale we undertook a preliminary theory driven confirmatory factor analysis (see S2 Appendix), which revealed 2 factors; (indirect) relational bullying characterized by verbal criticisms, and physical bullying (i.e. direct assault). This is in line with the suggestion of others that bullying comprises of relational and physical bullying [27,29,54,55]. As these two forms of bullying have been found to have a differential impact on the development of depression [27], and girls are more likely to report relational bullying[27,56], we examined their impacts separately in our analyses.

### Perceived family support

Family support was assessed at age 14 and 17 with the McMaster Family Assessment Device (FAD)-General Functioning Scale (FAD-GF[57], administered to adolescents. The FAD has adequate test-retest reliability, and differentiates between clinician assessed healthy versus unhealthy families[58]. The FAD-GF is a 12 item self-report questionnaire where respondents rate statements such as “*we can express our feelings to each other*” or “*there are lots of bad feelings in the family*”. The FAD-GF yields an estimate of overall family functioning[59]. In ROOTS, positive items on this scale were reverse coded so as to measure overall *negative* family functioning. To facilitate interpretation in our full model below, we inverted these scores so that a high score reflects *positive* family functioning (i.e. *family support*; scores ranged from -12 to -48 with a mean of -22.43 (SD = 5.76).

### Friendships: perceived quality of friendships

Friendships were assessed at age 14 and 17 with the self-report Cambridge Friendships Questionnaire (CFQ)[60]. The CFQ is an 8 item questionnaire assessing the number, availability, and quality of friendships (e.g. ‘Do you feel that your friends understand you?’, ‘are you happy with the number of friends that you’ve got at the moment’, ‘can you confide in your friends’, ‘do you have arguments with your friends that upset you?). The CFQ is derived from a semi-structured interview based on ethological principles of social relationships and the hypothesis that friendships are a key supportive network in the development of social and cognitive competencies [48]. The CFQ has good measurement invariance and external validity, and adequate test retest reliability across two week intervals (Kappa = .80)[61]. Scores in this sample ranged from two to 30 with a mean of 23.54 (SD = 4.21), and higher scores indicate better perceived overall quality of friendships (i.e. ‘*Friendships’*).

### Depressive symptoms

To assess current (last two weeks) depressive symptoms at age 14 and 17 we utilized the 33 item self-report Mood and Feelings Questionnaire (MFQ)[62] for eight to 18-year-olds. In this study, the MFQ has good internal consistency (α = 0.93), with sum scores ranging between zero and 57 with a mean of 13.6 (SD = 10.21), with higher scores indicating more depressive symptoms.

### Data

The raw data and analysis code used for this manuscript will be available from www.annelauravanharmelen.com/data/ and on figshare (https://figshare.com/authors/_/1376682).

### Statistical analyses

**Fig 1:**
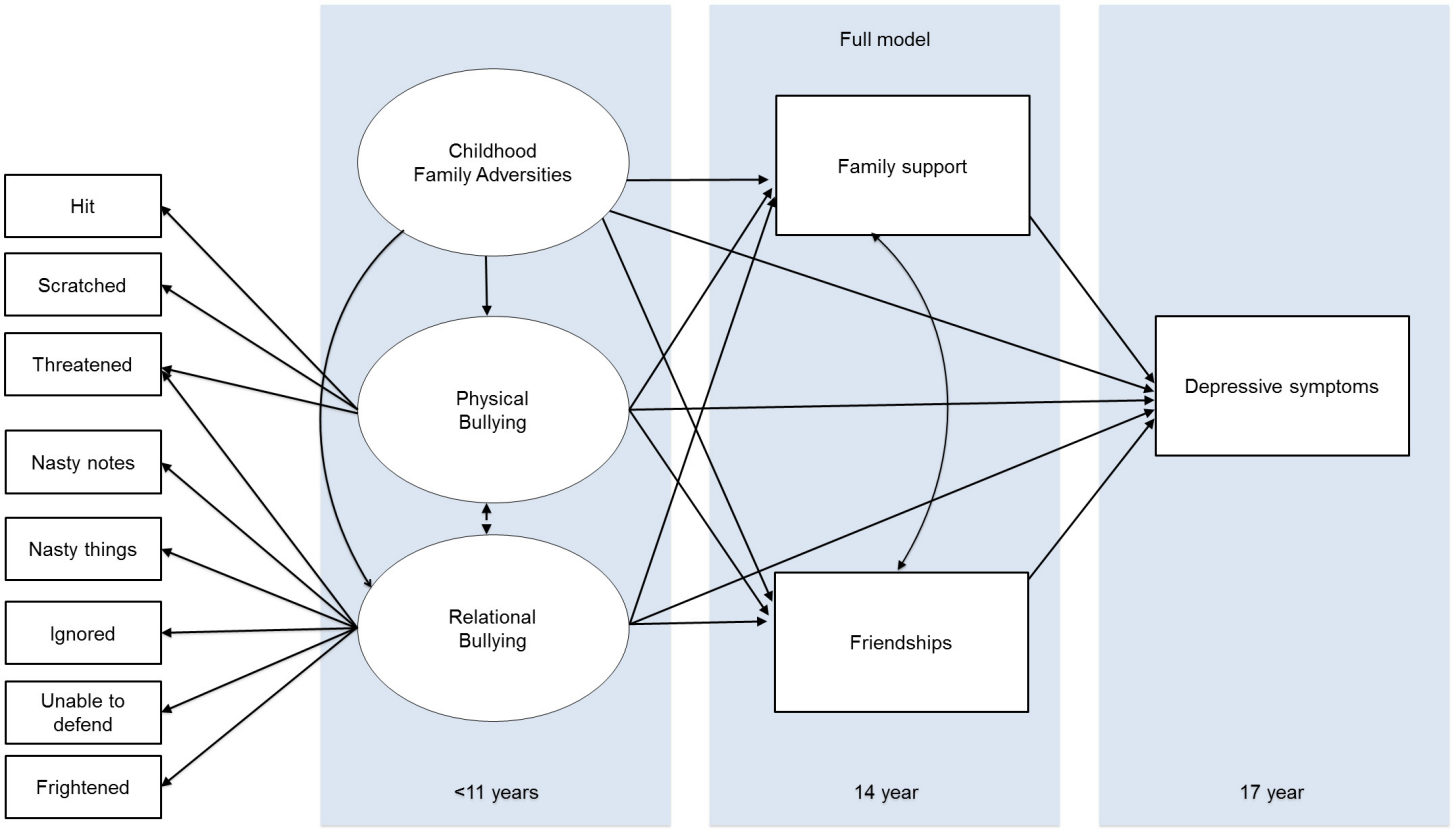
The full model based on the relationships in the literature and the bullying factor analysis. Note. Arrows depict hypothesized relationships. Black double headed arrows represent covariances that were specified between endogenous variables in the model. Black single headed arrows outside of the panels represent the factor loadings in the confirmatory factor analysis, whereas black single headed arrows inside the panels indicate regression paths. Gender was specified as covariate for all endogenous variables in this model, but is not depicted for simplicity.

We used Structural equation models (SEM) in Lavaan version 05.17[63] in R version 3.3[64] to examine the relations between family and friendships at age 14, and CFA and peer bullying before age 11, on depressive symptoms at age 17. Fig 1 displays the full theoretical model being tested in this study.

First, we specified the full structural equation model (Fig 1) for all participants who had complete data (N = 771). We specified gender as a covariate for all endogenous variables in this model. Note that within the model the latent variables ‘physical bullying’ and ‘relational bullying’ are estimated within the current sample. The latent CFA classes were based on our previous report[49]. Findings in this study remained the same when we used a binary CFA variable (i.e. Optimal vs. other classes (Discordant, Hazardous, and Aberrant). The latent variables are signified by the observed indicators shown in Fig 1. Relational and physical bullying (as well as friendships and family support) were allowed to co-vary within the model[7,31]. In posthoc analyses, we further tested the suggested mediations in the model using formal mediation analyses. In these mediation analyses we fitted a path analysis model in Lavaan that included the direct effect of X on Y and the indirect effect of X on Y via M. The standard errors for these defined parameters were computed using the Delta method[65], and the effect sizes were estimated using the method proposed by Iacobucci et al[66] (p. 153), namely as the proportion of the total effect (a*b+c’) explained by the mediation pathway alone (a*b) ranging from 0 to 100%. For posthoc moderation analyses, we fitted regressions whilst adding an interaction term that reflected the moderation.

Second we aimed to examine whether there were gender differences in the best fitting model. To do so, we fitted an equality constrained multi-group model, to examine whether the structural relations were identical in boys and girls.

**Fig 2:**
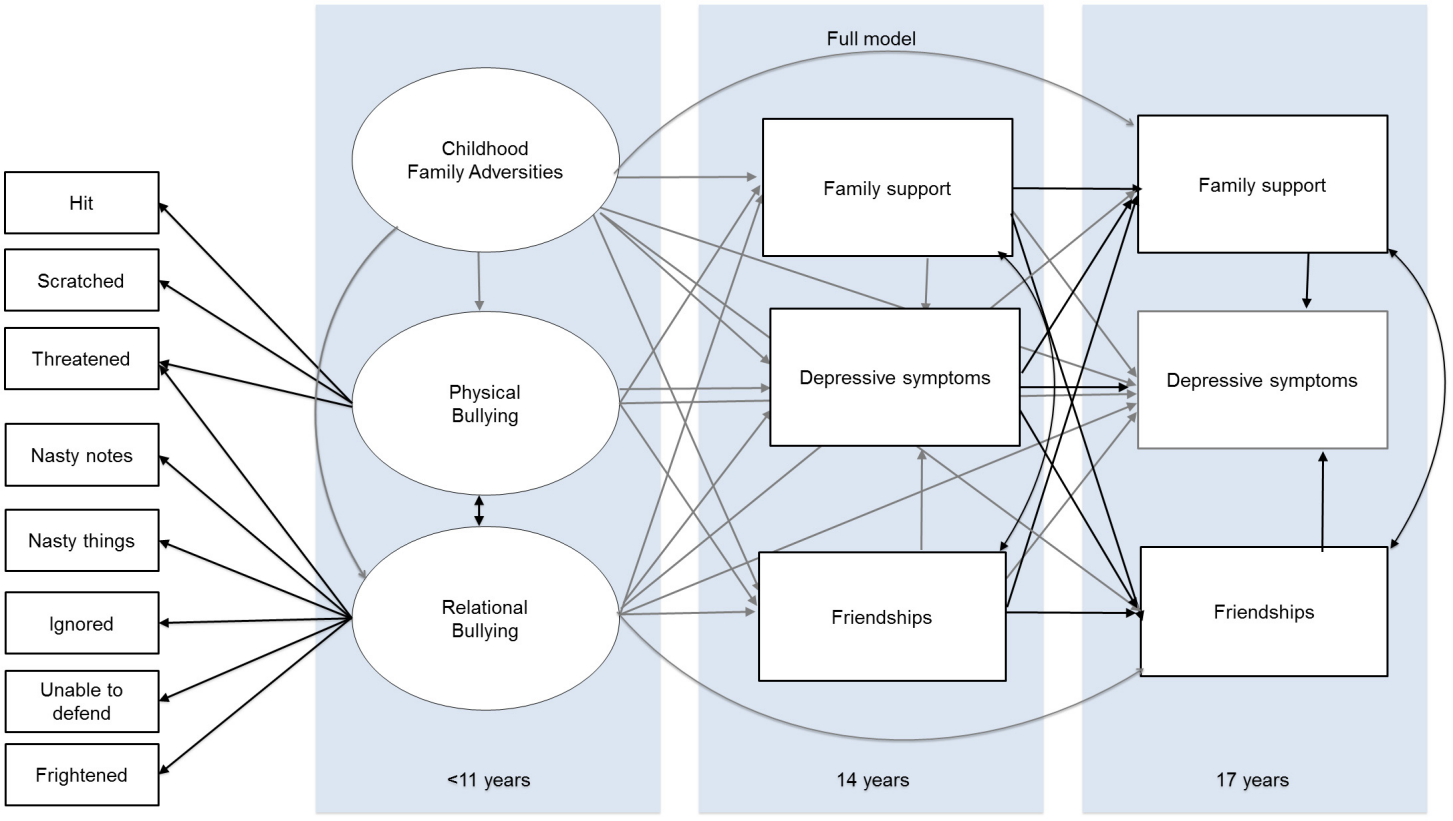
Model with temporal dimensions for friendships and/or family support. Note. For reasons of clarity grey arrows depict hypothesized relationship from 11 and 14 years, whereas black arrows represent hypothesized relationships from 14 and 17 years, and the factor loadings that were specified in the confirmatory factor analysis. Double headed arrows represent covariances that were specified in the model. Black single headed arrows outside of the panels represent the factor loadings in the confirmatory factor analysis, whereas black single headed arrows inside the panels indicate regression paths. Gender was specified as covariate for all endogenous variables in this model, but is not depicted for simplicity.

Third, we examined the temporal dimensions of adolescent support on depressive symptoms by including friendships, family support and depressive symptoms at both ages 14 and 17 (Fig 2). It is important to note that for our main hypothesis we fitted a model (depicted in Fig 1) that represented the current state of knowledge. However, in this analysis we fitted a more complex model that captured measurements of depressive symptoms, friendships and family support at ages 14 *and* 17 (see Fig 2), to see whether the influences shown above are consistent and to examine the temporal dynamics of these influences. In line with our previous analyses we again specified gender as a covariate for all endogenous variables, and relational and physical bullying as well as friendships- and family support at age 14 and 17 were allowed to co-vary within the model. It should be noted that this model is quite complex with 83 free parameters, and that despite the considerable sample size (i.e. complete data was available for N = 713) this is below an often-cited common guideline of N>10 per parameter[67]. This also indicates that division into genders and subgroups is, as opposed to the simpler model, not feasible.

In all analyses we used the option ‘mimic Mplus’ in Lavaan. We used the weighted least squares means and variance adjusted (WLSMV) estimator which is optimal for models that contain combinations of continuous, categorical and ordinal measures[68]. We modelled only complete cases and reported the Robust test statistic to account for deviations from normality of our data. As depressive symptoms, friendships and family support scores had different ranges, we scaled these variables to a standard normal distribution. We report chi-square (*X*^2^) fit statistics as well as the root mean squared error of approximation (RMSEA) and its 90% confidence interval (CI). RMSEA of less than 0.08 implies an acceptable model fit, and values of less than 0.05 imply a good fit[68]. Furthermore, we report comparative fit index (CFI), and the Tucker-Lewis index (TLI), where values of CFI &TLI >.95 represent good fit of the overall model[68]. We do not report SRMR value, as this is not defined for the WLSMV estimator in Lavaan in R. We used Chi-square comparisons to compare nested models.

## Results

### The importance of support after ELS on depressive symptoms

We first investigated the impact of friendships and/or family support at age 14 on depressive symptoms at age 17. The full model (Fig 1) had a good fit to the data *X*^2^ (48) = 175.602,p = .000,CFI = .992, TLI = .987, RMSEA = .059 (90%CI = .050-.068). To reveal the best model based on the known paths in the literature we next individually deleted the non-significant paths (based on highest P value). After every path deletion the model fit was re-evaluated. Table 2 depicts the specific paths that were deleted with every step.

**Table 2:** ROBUST model fit and model comparisons for each model.

	*X*^2^	DF	P	CFI	TLI	RMSEA	90% CI	Path that was removed	*X*^2^diff	Df diff	P
**First Model**	175.602	48	< .001	0.992	0.987	0.059	.50-.068	Family support ~ relational bullying			
**1**	168.938	49	< .001	0.993	0.988	0.056	.047-.066	Friendships~ physical bullying	0.01	1.00	0.91
**2**	165.853	50	< .001	0.993	0.989	0.055	.046-.064	Depressive symptoms~ CFA	0.07	2.00	0.97
**3**	164.816	51	< .001	0.993	0.989	0.054	.045-.063	Depressive symptoms~ relational bullying	0.70	3.00	0.87
**4**	162.352	52	< .001	0.993	0.99	0.052	.043-.062	Friendships~ CFA	1.53	4.00	0.82
**5**	162.297	53	< .001	0.993	0.99	0.043	.043-.061	Family support ~ physical bullying	3.95	5.00	0.56
**Final Model**	133.51	54	< .001	0.995	0.993	0.044	.034-.053		10.86	6.00	0.09

Note. CFA = Childhood Family adversities

**Fig 3:**
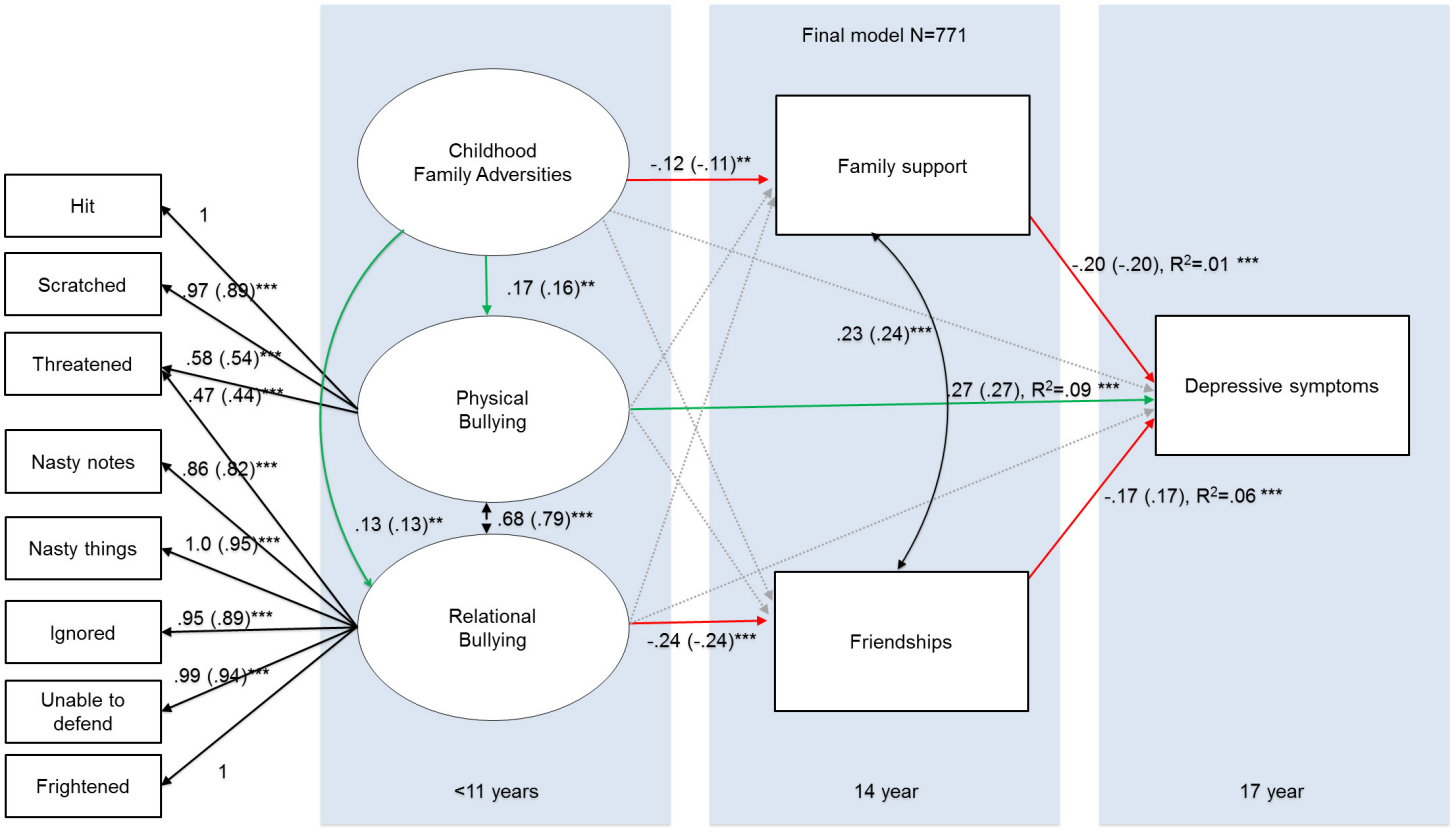
The influence of support after ELS on depressive symptoms. Note. *** = P < .001, ** = P < .01,* = P < .05. Estimates are unstandardized (standardized) path coefficients. Red arrows depict negative relationships, green arrows show positive relationships, Grey arrows depict non-significant (removed paths). Black double headed arrows represent covariance s that were specified between endogenous variables in the model. Black single headed arrows outside of the panels represent the factor loadings in the confirmatory factor analysis, whereas black single headed arrows inside the panels indicate regression paths. Gender was specified as covariate for all endogenous variables in this model, but is not depicted for simplicity.

The best fitting model (Fig 3) had a good fit [*X*^2^(54) = 133.51, p < .001, CFI = .99, TLI = .99, RMSEA = .044 (90% CI:.043-.053). This model shows two pathways through which symptom reduction at age 17 might occur. In the first path, CFA had a negative association with family support at age 14, which had a negative association with depressive symptoms at age 17. In the second path, CFA had a positive association with relational bullying, and relational bullying had a negative association with friendships at age 14, whereas friendships at age 14 had a negative relationship with depressive symptoms at age 17. Self-esteem may be an important confounding factor in the association between social support and depressive symptoms[69,70]. However, all findings and paths remained the same when self-esteem at age 14 (as measured with the Rosenberg self-esteem scale [71,72] was added to our model (see S3 Appendix for more information.

We next formally tested the proposed mediation paths in our model. A mediation analysis between CFA, depressive symptoms and family support suggested that the strength of the relationship between CFA and depressive symptoms was attenuated when taking family support into account. The indirect path (standardized estimate (Est) ab = 0.027(SE = .01), p = .005) explained 29% of the variance in the association between CFA and depressive symptoms (Est c = 0.094 (.04), p = .015), rendering the direct path between CFA and depressive symptoms non-significant (Est c' = 0.066 (= .04), p = .079).

Next, we tested the role of friendships as a mediator between relational bullying and depressive symptoms at age 17. This mediation analysis revealed that the indirect path from relational bullying to depressive symptoms through friendships (est ab = 0.023(0.007), p < .005) explained 35% of the variance of relationship between relational bullying and depressive symptoms (Est c = 0.17(0.024), p < .001), although the direct path between relational bullying and depressive symptoms remained significant (Est c' = 0.15(0.024), p < .001).

Our model also showed a strong association between CFA and relational bullying, whilst relational bullying was negatively associated with friendship support. A follow-up mediation analysis revealed that the indirect path from CFA to friendships through relational bullying (Est ab = -0.038(0.01), p < .001) explained 47% of the variance between CFA and friendships (Est c = -0.08(0.4), p = .04), and the direct path between CFA and friendships was non-significant (Est c’ = -0.04(.04), p = .26).

Our model also showed that CFA had a positive association with physical bullying, and physical bullying has a direct association with depressive symptoms at age 17 (but not with friendships or family support in adolescence). Indeed, a follow up mediation analysis revealed that the indirect path from CFA and depression (Est ab = 0.054(0.012), p < .001) explained 58% of the variance between CFA and depressive symptoms (Est c = 0.093(0.4), p < .001), rendering the direct path between CFA and depressive symptoms non-significant (Est c' = 0.039(0.04), p = .30).

In sum, our findings support a mediating role for friendships and family support in adolescence on depressive symptoms at age 17 after CFA and/or peer bullying. Our model also suggests that adolescents who reported to have experienced *physical* bullying (in isolation or together with CFA) showed no mediating effects of family support or friendships on subsequent depressive symptoms.

### Social environment as moderator

Our model represents, in essence, a mediation of positive social family and friendship environments across adolescence. However, one may also hypothesize a moderating effect of social support, where friendships reduce the effect of relational bullying on depressive symptoms at age 17, and/or family support reduces the strength of the association between CFA and later depressive symptoms. Therefore, we next tested these hypothesize moderations. In a model where family support moderated the relationship between abuse and depressive symptoms at age 17 we found that there was no significant relation between CFA and depressive symptoms at age 17 (Est = -0.04, SE = .03, t = -1.09, p = .28), and there was a significant relationship between family support and depressive symptoms at age 17 (Est = -0.24, SE = .03, t = -6.91, p < .001). However, there was no significant moderation (CFA*family support) on depressive symptoms at age 17 (Est = 0.02, SE = .03, t = 0.54, p = .59). Next we tested whether friendships moderated the relationship between relational bullying and depressive symptoms at age 17. There was a significant relation between relational bullying and depressive symptoms at age 17 (Est = 0.34, SE = .05, t = 6.62, p < .001), and there was a significant relationship between friendships support and depressive symptoms at age 17 (Est = -0.22, SE = .04, t = -5.10, p < .001), however, there was no significant moderation (relational bullying*friendship) on depressive symptoms at age 17 (Est = 0.04, SE = .04, t = 0.97, p = .33). The lack of moderating effects contrasts markedly with the indirect pathways that were proposed in our model, and are in line with our findings of mediating roles for friendship and family support.

### Sex differences

Next, we examined whether the effects reported above for the full sample differed between boys and girls. Examining the descriptive statistics, Self-reported relational bullying, friendships, and family support did not differ between boys and girls (Table 3). However, there were more boys in the hazardous CFA group, and boys reported more physical bullying, whereas girls reported more depressive symptoms at age 17.

**Table 3:** Characteristics for boys and girls separately.

	**Boys (N = 322)**	**Girls (N = 449)**		**Wilcox t**	**P**
**CFA (#O/A/D/H)**	228/22/53/19	330/30/75/14		52003	< .001
**% O/A/D/H**	70.81/6.83/16.46/5.90	73.49/6.68/16.70/3.11			
	**Mean (SD)**	**Mean (SD)**	**t**	**DF**	**P**
**Relational bullying**	0.35 (.69)	0.39 (0.67)	-0.86	680.16	0.39
**Physical bullying**	0.41(0.68)	-0.10 (0.63)	10.49	656.62	< .001
**Friendships**	23.73 (4.26)	23.54 (3.93)	0.64	657.48	0.52
**Family support**	-22.15 (5.41)	-22.23 (6.01)	0.21	730.30	0.84
**Depressive symptoms at age 17**	11.45 (9.24)	15.14 (10.60)	-5.14	740.20	< .001

Note. CFA = Childhood Family adversities, O = optimal parenting, A = aberrant parenting, D = discordant parenting & H = Hazardous parenting.

To formally test for possible differences in the structural relations between boys and girls, we fitted a multi-group model (322 boys, 449 girls), using the same ‘*final’* model (same regression paths as depicted in Fig 3; whilst removing gender as covariate). This model showed good fit to the data *X*^2^ (126) = 204.74,P = .001, CFI = .995, TLI = .995, RMSEA = .040 (90% CI: .030-.050).

Next, we re-ran this final model, only now with equality constraints. We first fixed the factor loadings to be equal to ensure measurement invariance, cf.[73]; a ‘*factor loadings equality constrained model*’ that showed good fit [*X*^2^ (103) = 157.009,P = .000,CFI = .997, TLI = .996, RMSEA = .037 (90% CI: .025-.048)]. We then also fixed the regressions to be equal, thereby implying that all regression relationships drawn in Fig 3 should be equal in both groups (i.e. boys and girls). This ‘*factor and regressions equality constrained’* model also had good fit to the data[*X*^2^ (110) = 170.398,p = .000,CFI = .996, TLI = .996, RMSEA = .038 (90%CI: .026-.049)]. We then compared the ‘*factor loadings and regressions equality constrained’* to the ‘*factor loadings equality constrained model*’, thereby examining if the regressions should be allowed to differ between the genders. The chi-square model comparison showed that the more complex ‘*factors equality constrained model*’ fitted the data significantly better (*X*^2^diff (7) = 14.29, p = .046), suggesting that the regression paths are different in boys compared to girls.

**Fig 4:**
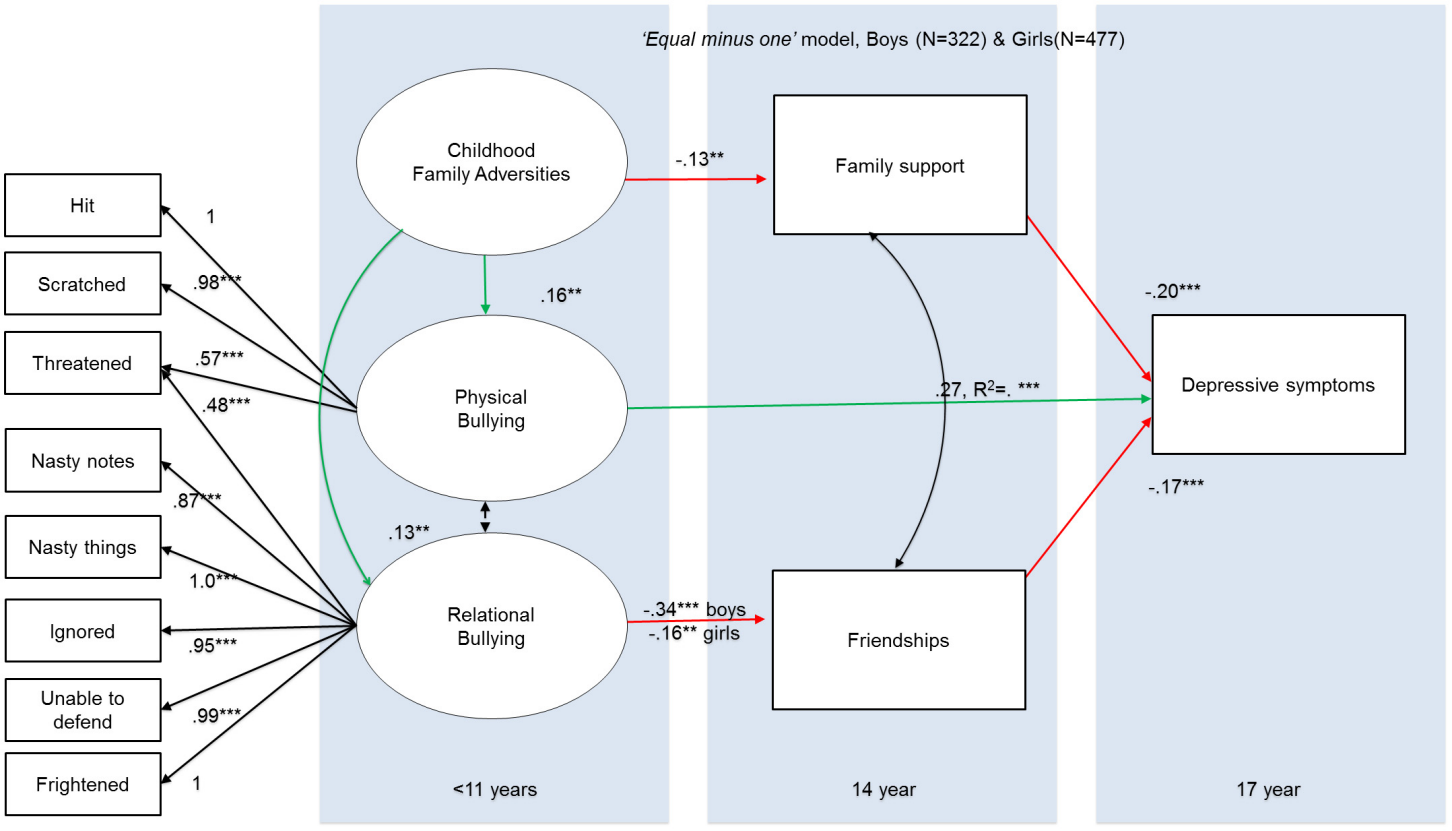
Best fitting model for boys and girls: The factors and regressions equality minus one constrained (‘equal minus one’) model. Note. *** = P < .001, ** = P < .01,* = P < .05. Estimates are unstandardized path coefficients in boys and girls. Red arrows depict negative relationships, green arrows show positive relationships. Black double headed arrows represent covariance’s that were specified between endogenous variables in the mode. Black single headed arrows outside of the panels represent the factor loadings in the confirmatory factor analysis, whereas black single headed arrows inside the panels indicate regression paths.

To examine which paths were significantly different in boys vs. girls, we then examined modification indices for the ‘*factors and regressions equality constrained’* model. This inspection showed that the association between *relational bullying* and *friendships* had the largest modification index (mi = 11.45 (1), p = 0.0007). Examination of the path coefficients in the ‘*factors equality constrained model*’ (where the regression paths were allowed to differ) revealed that this relationship was *more negative* in boys (est = -0.345, SE = 0.058, Z = -5.930, p < .000) than in girls (Est = -0.160, SE = 0.048, Z = -3.327, p< 0.001). We now re-ran the ‘*factors and regressions equality constrained’* whilst now also specifying that this one path should be allowed to differ. This ‘*factors and regressions equality minus one constrained’* model had good fit to the data [*X*^2^ (109.00) = 153.644, p = .003,CFI = .997, TLI = .997, RMSEA = .033 (90%CI: .019-.044)]. Finally, we compared this ‘*factors and regressions equality minus one constrained’* model with the ‘*factors equality constrained model*’ (i.e. all regression paths were allowed to differ), thus testing whether a model where only one path differs between boys and girls is significantly different from a model where all paths differ between boys and girls. This analysis showed that the more complex ‘*factors equality constrained model*’ (i.e. all regression paths were allowed to differ), did not fit the data significantly better than the ‘*factors and regressions equality minus one constrained’* model (*X*^2^diff (6) = 3.6455, P = 0.7245). Therefore, the ‘*factors and regressions equality minus one constrained’* is preferred for our data, and depicted in Fig 4. In this model, no differences between boys and girls were found (i.e. similar paths coefficients for boys and girls) except for the path between relational bullying and friendships, which was more negative in boys.

### Developmental influences of friendships and/or family support

**Fig 5:**
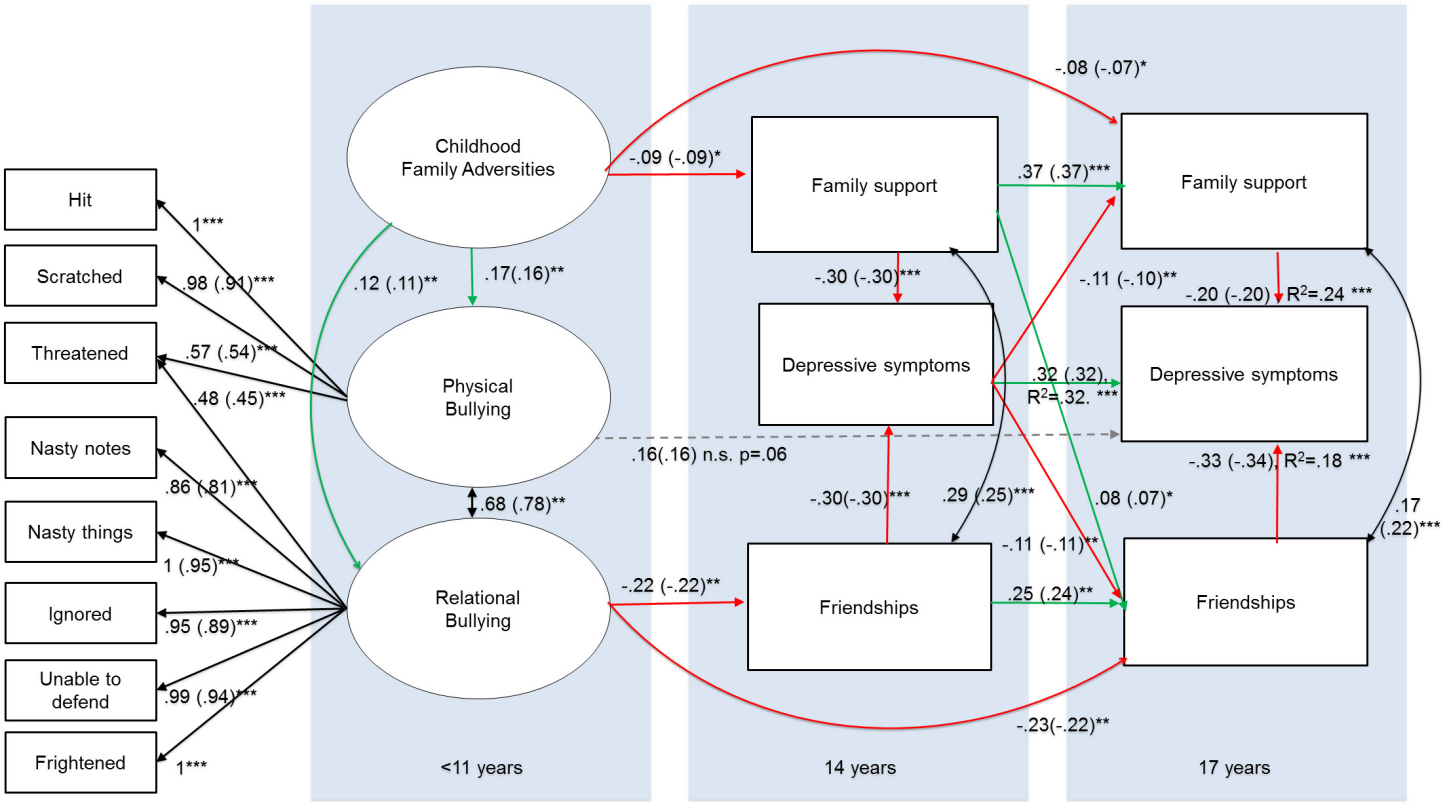
Developmental influences of friendships and/or family support. Note. *** = P < .001, ** = P < .01,* = P < .05, n.s. = not significant. Estimates are unstandardized (standardized) path coefficients. Red arrows depict negative relationships, green arrows show positive relationships. Grey dashed represents a non -significant relationship. Black double headed arrows represent covariance’s that were specified between endogenous variables in the model. Black single headed arrows outside of the panels represent the factor loadings in the confirmatory factor analysis, whereas black single headed arrows inside the panels indicate regression paths. Gender was specified as covariate for all endogenous variables in this model, but is not depicted for simplicity.

We next investigated the developmental influences of friendships and/or family support on depressive symptoms at age 17 through testing a model that included friendships, family support and depressive symptoms at ages 14 and 17 (as hypothesized in Fig 2). This full model had a good fit to the data *X*2 (66) = 184.770, p = < .001, CFI = .99, TLI = .99, RMSEA = .050 (90% CI = .042-.059). The fitted model is shown in Fig 5 and shows that friendships and/or family support in adolescence indirectly acted on depressive symptoms at age 17 through depressive symptoms at age 14, and through friendships and/or family support at age 17. These findings were confirmed when we re-ran the model whilst specifying these indirect effects: we found support for indirect effects of family support at age 14 on depressive symptoms at age 17 through family support at age 17 (Est = -.07,SE = .01, Z = -5.55 P < .001), and through depressive symptoms at age 14 (Est = -.09,SE = .01, Z = -8.12 P < .001). Friendship support at age 14 had an indirect effect on depressive symptoms at age 17 through friendship support at age 17 (Est = -0.08 SE = .01,Z = -6.16 P < .001), and through depressive symptoms at age 14 (Est = -.04,SE = .01, Z = -2.87 P = .004). Interestingly, the model also suggested that family support at age 14 was positively related with friendships at age 17. Finally, another interesting finding from this model is that depression at age 14 is negatively associated with both friendship and family support at age 17.

## Discussion

Our study suggests that friendships and/or family support in adolescence may reduce depressive symptoms in boys and girls who have been exposed to early life stress (childhood family adversity and/or relational bullying before age 11). Our findings suggest a cascading effect of ELS on later life. We find support that CFA is associated with bullying from peers (both relational and physical bullying), and that CFA is associated with reduced adolescent family support. Furthermore, we find that peer relational bullying is associated with reduced friendship support in adolescence, and that relational bullying mediates 47% of the negative association between CFA and friendship support (corroborating[29]).

Crucially, despite these cascading negative effects of ELS on later interactions, our findings also suggest that *positive* social environments in adolescence may reduce depressive symptoms in later life. Specifically, our findings suggest two pathways through which a positive social environment might mediate the link between ELS and depressive symptoms in later life. First, CFA was associated with reduced perceived *family support*, which has a negative association with depressive symptoms in boys and girls (corroborating[35]). In the second pathway relational bullying was associated with reduced self-reported *friendships* at 14 years, which were negatively associated with depressive symptoms at age 17 in boys and girls (corroborating[35,42,43]). Follow up mediation analyses confirmed these results, and we found no support for moderating effects of the social environment on depressive symptoms. Finally, when we tested the temporal dynamics of friendships and family support, we found that friendships and/or supportive families in early adolescence indirectly affect depressive symptoms at age 17 through intermediate effects on depressive symptoms at age 14, and through intermediate effects on friendships and/or family support at age 17. In sum, our findings suggest that adolescent family and friendships support may reduce later depressive symptoms in adolescents with a history of CFA and/or peer relational bullying.

The negative association between relational bullying and adolescent friendships was stronger in boys when compared to girls, which is in line with findings that the association between relational bullying and depressive symptoms appears to be stronger for boys compared to girls[27]. Perhaps boys reported more *severe* relational bullying events, or it may be that boys are more sensitive to relational bullying. In addition, our model suggests that strong links between CFA and both relational and physical bullying, which is in line with findings that CFA predicts greater likelihood of further peer bullying (e.g[7]), and this double disadvantage is related with more severe depressive symptoms[30]. In sum, our findings suggest that mental health interventions aimed at enhancing family support and peer relationships may be particularly helpful for adolescents who were exposed to CFA and/or peer relational bullying. Of note is that our findings suggest that such strategies may not be beneficial for those exposed to physical bullying.

This study finds evidence for several key pathways through which the adolescent social environment affects later life mental health. However, the *mechanism* through which social support exerts its influence on depressive symptoms remains unknown. It has been suggested that supportive family environments may increase resilience through enhancing coping performance and reducing threat appraisals [69], through a positive effect on self-esteem, self-regulation and through offering stress-relief[33,38,74]. Another way in which family support may increase resilience is through modelling effective interpersonal skills[37]. The mechanisms through which friendship support may increase resilience are similarly not well understood. Perceived friendship support may have a similar positive effect on coping skills, self-esteem and threat appraisal[33], perhaps through companionship, and experiences of low conflict[69]. There are indications that friendships increase adolescents perceived friendship self-efficacy; their beliefs that they are able to communicate and engage with their friends, to resolve conflict and to manage their interpersonal emotions[42]. Indeed, friendship self-efficacy is negatively associated with depressive symptoms in boys and girls[42]. Another way in which friendship may increase resilience is through updating self-cognitions. It is well established that CFA and relational bullying each induce negative self-cognitions[22,47,75,76], which have been linked to subsequent depression (e.g.[76–78]). Perhaps positive friendships and family support in adolescence provide opportunities for a more positive update of self-cognitions. Physical bullying is not as strongly related to negative self-cognitions[27], which may explain why social support was not effective in adolescents that experienced physical bullying. Examining the mechanisms of action for adolescent friendships and family support is an important avenue for future research.

To our knowledge, this is the first study in a community sample of adolescents that simultaneously examines the relations between ELS, adolescent friendships and/or family support, and later depressive symptoms. However, this study is not without limitations. First, other factors such as genotypes[29] or temperament [79] may influence the paths in the model. However, these factors were not part of the theoretical framework addressed in this study, and recent reports on this cohort suggest that temperament is not likely to alter the current findings[79]. Second, bullying in our sample was retrospectively assessed at age 17, and self-report measures of bullying may be sensitive to recall bias. However, inflated recall of bullying in depressed adolescents would likely only further reduce the already non-significant relationship in our model between relational bullying and depression at 17. Furthermore, retrospective self-reported bullying has been found to have adequate accuracy and reliability[80]. Third, our cohort is more affluent compared to UK norms[51], therefore caution should be taken when generalizing our findings. Fourth, CFA was assessed from the primary care-giver, which may have led to an underreporting of CFA. Although an underreporting of CFA would reduce the reported relationships in our model, indicating that the *actual* relationships between care-giver assessed CFA and adolescent assessed family support, peer bullying, or friendships are stronger. Importantly, underreporting of CFA would not impact on the relationships between adolescent friendships and/or family support and depressive symptoms at age 17.

## Conclusion

First, our study finds support for a prolonged negative impact of CFA on adolescent family and peer interactions and consequently on depressive symptoms in late adolescence. These findings stress the importance of early intervention and prevention programs[81–83]. For instance, case management services for families at risk[81], parenting programs [84] and the Nurse-Family partnership program [82] may be effective in reducing the occurrence of CFA. Second, our study suggests that friendships and/or family support in adolescence may attenuate subsequent depressive symptoms in boys and girls exposed to CFA and relational bullying. Clearly, our study stresses the importance of a positive social environment in early life and in later adolescence. These findings have important implications, for instance they could inform psychosocial education programs that discuss the prolonged effects of childhood maltreatment and peer relational bullying and how to counter these effects. These psychosocial programs may be run in schools, general health medical centers, mental health institutions, hospitals, sports clubs, and other institutions relevant for psychosocial education. In addition, our study suggests the need for interventions aimed at increasing the positive social environments in the adolescent epoch. For instance, school based mental health interventions aimed at finding and sustaining friendships through the active facilitation of social skills training in victimized boys and girls[85] may increase mental health resilience in adolescents that experienced relational bullying (with or without concurrent CFA). Similarly, our findings suggest that the efficacy of interventions that increase positive parenting, and video feedback programs[86] or the triple P intervention programs[87] might be fruitful for the parents of adolescents who have experienced early life CFA. Our study suggests that such interventions aimed at increasing friendships and family support may have great potential to reduce late adolescent depressive symptoms in boys and girls that experienced early life child maltreatment and/or relational bullying.

## Supporting Information

S1 Appendix Copy of the Peer Victimization Questionnaire. (DOCX)

S2 Appendix Factor analysis for the Peer Victimization Questionnaire (PVQ). (DOCX)

S3 Appendix Additional Self-esteem analysis. (DOCX)
