# Supplementary material for: Friendships and Family Support Reduce Subsequent Depressive Symptoms in At-Risk Adolescents
Source: PLoS One. 2016 May 4;11(5):e0153715. doi: 10.1371/journal.pone.0153715 (PMC4856353; doi:10.1371/journal.pone.0153715)
Supplement: S1 Appendix — (DOCX) [file pone.0153715.s001.docx]

## S1 Copy of the Peer Victimization Questionnaire

### This is a short questionnaire about bullying at primary school. First, please answer the two questions in bold. If the answer to either is ‘yes’, answer the short questions which follow ticking the boxes which best describe how often each thing happened. If the answer to either of the main questions is ‘no’ leave the short questions blank. If there was more than one period of bullying, please answer for the first time, when you were youngest. Thank you

|  | Never | Once | Occasionally/rarely | Weekly | Daily/almost daily |
| --- | --- | --- | --- | --- | --- |
| 1. At primary school, aged 5-11, I was bullied by other children/another child -(if yes carry on, if no, move to Q2) |  |  |  |  |  |
| - I was hit, punched or kicked |  |  |  |  |  |
| - I was scratched |  |  |  |  |  |
| - I was threatened |  |  |  |  |  |
| - I was sent nasty notes/texts/emails |  |  |  |  |  |
| - I was ignored |  |  |  |  |  |
| - People said very nasty things to me |  |  |  |  |  |
| - I felt unable to defend myself |  |  |  |  |  |
| - I was frightened |  |  |  |  |  |
| How old were you the first time ……………….…About how long did it last? *(please circle nearest*) it only happened once / it lasted: weeks / months / years | | | | | |
