# Supplementary material for: Friendships and Family Support Reduce Subsequent Depressive Symptoms in At-Risk Adolescents
Source: PLoS One. 2016 May 4;11(5):e0153715. doi: 10.1371/journal.pone.0153715 (PMC4856353; doi:10.1371/journal.pone.0153715)
Supplement: S2 Appendix — (DOCX) [file pone.0153715.s002.docx]

## S2 Factor analysis for the Peer Victimization Questionnaire (PVQ)

### Bullying victimization from peers can be divided in to two components; direct physical (e.g. hitting, scratching), and more indirect relational bullying (e.g. ignoring, threatening, sending nasty texts)[17,27,63,64]. Therefore, we expected that the PVQ would give a two-factor structure (relational and physical bullying). For this reason, we examined the structure the PVQ using a theory-driven Confirmatory Factor Analysis (CFA) in N=900 participants who had complete PVQ data. We hypothesized that the items “*I was hit, punched, kicked*”, and ‘*I was scratched*’ would load onto a physical bullying latent variable, whereas the items “*I was sent nasty notes/text/emails*”, ‘*I was threatened*”, “*I was ignored*”, “*people said nasty things to me*”, “*I felt unable to defend myself*”, and “*I was frightened*” would load onto a relational bullying latent variable. The model fit showed good fit to the data when the item ‘*I was threatened’* was allowed to load on both factors [N=900, *X*^2^(18)=58.617,P=.000, CFI=.998,TLI=.996,RMSEA=.050 (90% CI=0.036-0.065) see Table S1 for the specific factor loadings]. We implemented this two factor measurement model in our full SEM.

### Table S1, Factor loadings for the Peer Victimization Questionnaire items.

| **Latent variables:** | **Items:** | **CFA without cross loading** | | | | **CFA with cross loading** | | | |
| --- | --- | --- | --- | --- | --- | --- | --- | --- | --- |
|  |  | Est. | (SE) | Z | P(>\|z\|) | Est. | (SE) | Z | P(>\|z\|) |
| **Relational bullying** |  |  |  |  |  |  |  |  |  |
|  | I was frightened | 1.00 |  |  |  | 1.00 |  |  |  |
|  | I felt unable to defend myself | 1.01 | (0.01) | 79.99 | <.001 | 1.00 | (0.01) | 79.98 | <.001 |
|  | I was ignored | 0.94 | (0.02) | 64.61 | <.001 | 0.94 | (0.02) | 64.77 | <.001 |
|  | I was sent nasty notes/texts/emails | 1.01 | (0.01) | 86.39 | <.001 | 1.01 | (0.01) | 85.25 | <.001 |
|  | I was threatened | 0.80 | (0.03) | 27.04 | <.001 | 0.48 | (0.05) | 9.83 | <.001 |
|  | People said nasty things about me | 0.96 | (0.02) | 64.74 | <.001 | 0.80 | (0.03) | 27.16 | <.001 |
| **Physical bullying** |  |  |  |  |  |  |  |  |  |
|  | I was hit, punched or kicked | 1.00 |  |  |  | 1.00 |  |  |  |
|  | I was scratched | 0.97 | (0.03) | 30.05 | <.001 | 0.98 | (0.03) | 31.23 | <.001 |
|  | I was threatened |  |  |  |  | 0.58 | (0.06) | 10.12 | <.001 |
