# Supplementary material for: Friendships and Family Support Reduce Subsequent Depressive Symptoms in At-Risk Adolescents
Source: PLoS One. 2016 May 4;11(5):e0153715. doi: 10.1371/journal.pone.0153715 (PMC4856353; doi:10.1371/journal.pone.0153715)
Supplement: S3 Appendix — (DOCX) [file pone.0153715.s003.docx]

## S3 Additional self-esteem analysis

We were interested in investigating whether the effects of friendship support and/or family support were explained by self-esteem at age 14 (measured with the Rosenberg self-esteem scale[68,69]), we added a path where friendships, family support, and physical bullying were related with self-esteem at age 14. We also added a path where self-esteem at age 14 was associated with depressive symptoms at age 17. This model has a good fit, X2(63)=190.18,p=.000,CFI=.992, TLI=.988, RMSEA=.052 (90%CI=.043-.06), and all the existing paths in the model remained (including the associations between family support and depressive symptoms and friendship support and depressive symptoms, see below Fig S1 for details of the paths). In addition, friendship support and family support have a positive association with self-esteem at age 14, and self-esteem has a negative association with depressive symptoms at age 17.


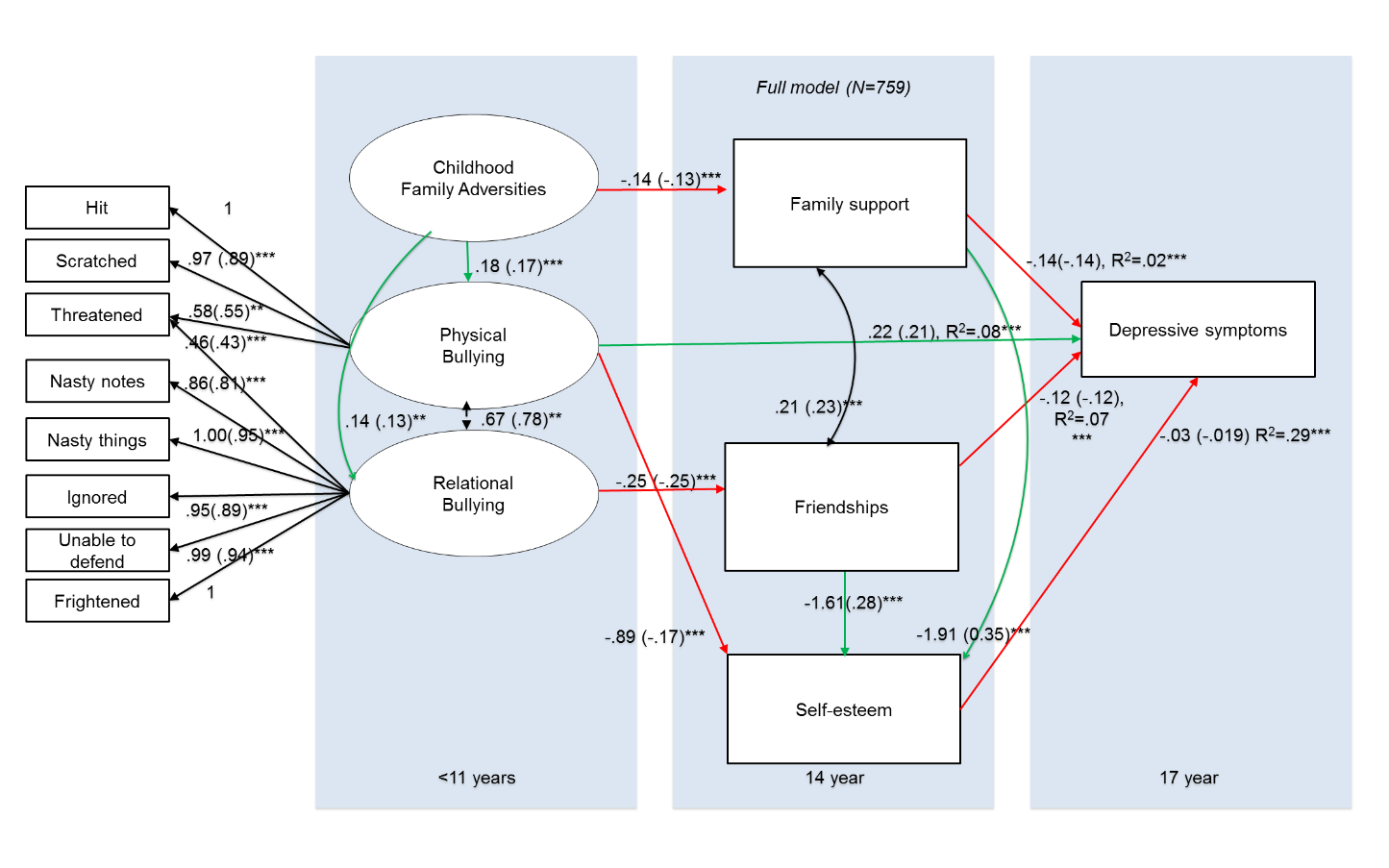


### Figure S1. SEM with Self-esteem at age 14.

*Note. ***=P<.001, **=P<.01,*=P<.05. Estimates are unstandardized (standardized) path coefficients. Red arrows depict negative relationships, green arrows show positive relationships. Black double headed arrows represent covariance s that were specified between endogenous variables in the model. Black single headed arrows outside of the panels represent the factor loadings in the confirmatory factor analysis, whereas black single headed arrows inside the panels indicate regression paths. Gender was specified as covariate for all endogenous variables in this model, but is not depicted for simplicity.*
